# Uncovering morphometric, germination, and genetic divergence patterns in *Euterpe edulis* for breeding and conservation applications

**DOI:** 10.1038/s41598-025-02606-7

**Published:** 2025-09-26

**Authors:** Guilherme Bravim Canal, Marcello Zatta Péres, Francine Alves Nogueira de Almeida, Maurício dos Santos Araújo, Jéssica Tetzner de Oliveira, Jônatas Gomes Santos, Gilza Barcelos de Souza, Marcia Flores da Silva Ferreira, Rodrigo Sobreira Alexandre, Adésio Ferreira

**Affiliations:** 1 Department of Agronomy, Federal University of Espírito Santo, Alegre, Espírito Santo Brazil; 2 Department of Forest and Wood Sciences, Federal University of Espírito Santo, Jerônimo Monteiro, Espírito Santo Brazil; 3 Genetics Department, University of São Paulo, Genetic Diversity and Plant Breeding Laboratory, Piracicaba, SP Brazil

**Keywords:** Plant breeding, Conservation genomics

## Abstract

*Euterpe edulis* exhibits significant ecological, social, and economic potential, comparable to *E. oleracea*, for both the fresh fruit pulp market and industrial applications as a natural pigment. The commercial management of *E. edulis* fruits is economically promising; however, further studies are required to support improvement, conservation, and propagation programs. In this study, we aimed to assess the genetic control of key traits by estimating variance components, genetic parameters, and the associative relationships between fruit morphometric traits, seed characteristics, and seedling emergence using Pearson correlation and path analysis. Additionally, we quantified the genetic divergence among *E. edulis* accessions. Thirteen phenotypic traits and eight microsatellite markers were analyzed in 72 genotypes from different origins. High heritability values (ranging from 0.75 to 0.99) were observed for fruit, seed, and emergence traits, whereas morphophysiological growth traits exhibited lower heritability estimates. Genetic correlations of up to 0.88 were detected among fruit morphometric traits. Seedling basal diameter and leaf area had direct positive effects on the seedling quality index, while seedling height exerted a direct negative effect. Genetic divergence analysis demonstrated the efficiency of sampling genotypes from different origins to preserve the species’ genetic variability. These findings provide insights to guide the establishment of germplasm collections in the field, maximizing the potential for genetic recombination among divergent genotypes and offering a solid foundation for future studies employing expanded molecular resources to explore trait architecture more deeply.

## Introduction

The Atlantic Forest, the second-largest forest in South America, is one of the most threatened and species-rich biomes in the world^1^ and is considered one of the 35 biodiversity hotspots on the planet^2^. Due to its remarkable biodiversity, protecting this biome is essential for the conservation of threatened species and their interactions within the ecosystem^3^. Among its vast array of plant genetic resources, fruit-bearing palms play a crucial ecological role by providing food during periods of scarcity in the forest^4^, particularly those of the genus *Euterpe*. Notably, *E. edulis* Mart. stands out^5^. Commonly known as the juçara or juçaizeiro palm, this species holds significant ecological, cultural, and economic value^6^. However, human pressures on its natural resources, combined with the fragmentation of the biome, have led to its classification as a threatened species^7,8^.

The juçara palm (*E. edulis*) exhibits significant economic potential through the sustainable management of its fruits, in addition to its crucial ecological role. The species has the capacity to support a production chain analogous to that of the açaí palm (*E. oleracea*)^9,10^. The fruits of the juçara palm possess high nutraceutical value and substantial potential as a functional food, mainly associated with their high anthocyanin content^11^. Furthermore, the pulp exhibits nutritional characteristics comparable to or superior to those of açaí pulp (*E. oleracea*)^12–14^. However, the commercial exploitation of juçara requires the establishment of cultivated orchards to ensure economic viability. Industrial processors in the state of Espírito Santo report that the current supply of juçara fruits is insufficient to meet market demand. Moreover, the production potential is constrained by fruit availability, which is predominantly sourced through sustainable management in native forest fragments. To advance the commercial cultivation of the species, the development of superior genetic materials through breeding programs is essential. Such programs rely initially on a breeding foundation population with high genetic diversity and a thorough understanding of the genetic control of key traits, which are critical for defining effective strategies within the breeding program.

The estimation and prediction of genetic parameters and variance components in perennial species are typically conducted using the REML/BLUP method (restricted maximum likelihood/best linear unbiased prediction)^15^. In particular, for species of the genus *Euterpe*, this approach mitigates challenges associated with data collection, such as imbalance and the absence of a structured experimental design. By enabling the correction of observations, the REML/BLUP method enhances the accuracy of variance component estimates^16^, thereby improving the understanding of the genetic control of key traits.

Within breeding programs and seedling nursery practices, understanding the relationships between phenotypic traits and genetic diversity is fundamental for the effective conservation of genetic resources and the development of superior genotypes. Assessing genetic diversity is crucial to prevent the use of genetically similar materials, thereby reducing the risk of allele loss due to inbreeding. Consequently, analyses of both phenotypic and molecular diversity play a critical role in genotype characterization and selection, enhancing the efficient utilization of available genetic resources, ensuring the long-term sustainability of breeding programs, and contributing to the successful conservation of species’ genetic resources. In this context, the objective of this study was to investigate genetic control through the estimation of variance components and genetic parameters, as well as the associative relationships among fruit and seed morphometric traits, seedling emergence, and early development. Additionally, this study aimed to quantify the genetic divergence within *E. edulis* germplasm, providing essential information to support the establishment of *ex situ* germplasm collections.

## Material and methods

### Plant material

**Fig. 1:**
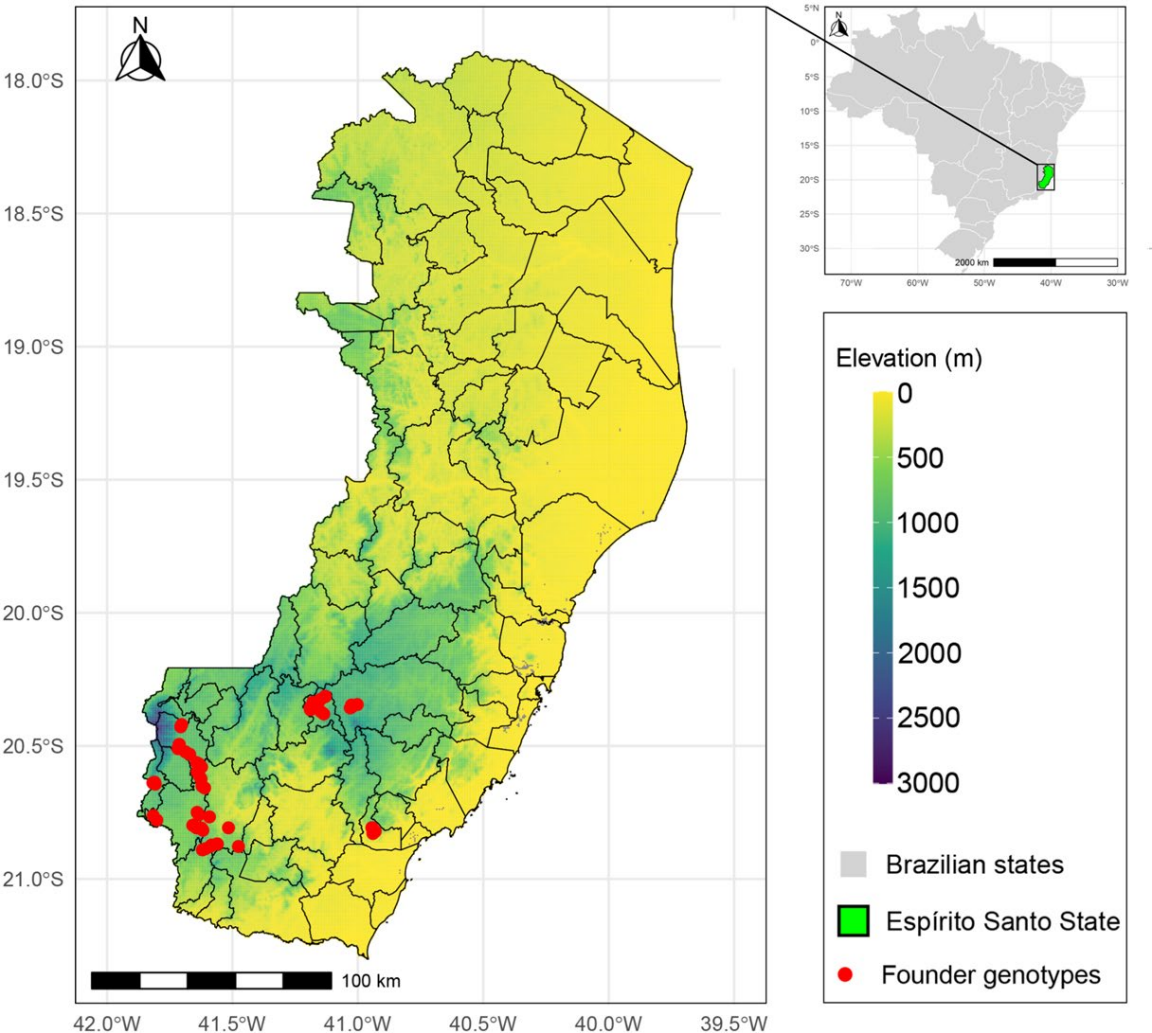
Geographic location of sampled *E. edulis* founder genotypes, indicated by red dots, in the state of Espírito Santo, Brazil. Map generated using the R software.

Fruits from 72 *E. edulis* trees were sampled from fragments of the Atlantic Forest located on private properties across eight municipalities in the state of Espírito Santo, Brazil: Alegre, Domingos Martins, Dores do Rio Preto, Guaçuí, Ibitirama, Rio Novo do Sul, São José dos Calçados, and Venda Nova do Imigrante (Fig. 1). The selection criteria for the trees included a minimum distance of 150 meters between sampled individuals, good phytosanitary and physiological conditions, and superior fruit production potential compared to neighboring trees. The selected trees were located at altitudes ranging from 500 to 1500 meters. Voucher specimens of *E. edulis* were collected in Espírito Santo: VIES039447^17^, VIES038826^18^, and MBM00422915^19^. The specimens were deposited in the herbaria VIES (Universidade Federal do Espírito Santo) and MBM (Museu Botânico Municipal). Voucher information and high-resolution images are available through the Reflora Virtual Herbarium. All collections were conducted with prior consent from landowners and duly authorized by the Brazilian Ministry of the Environment through the Chico Mendes Institute for Biodiversity Conservation (ICMBio/SISBIO) (Decisions n.o 59344-2 and 87764-3), following national regulations for scientific research.

Fruits were collected at full maturation, characterized by a blackish pulp color. Additionally, a fragment of the stipe cortex from each founder genotypes was collected, placed in Kraft paper bags containing silica gel, properly labeled, and transported to the Laboratory of Genetics and Plant Breeding (LGMV) at the Federal University of Espírito Santo (UFES), Alegre campus. Samples were stored at -80 $^{\circ }$C for 24 hours before being lyophilized for 72 hours.

### Experimental conduction and phenotyping

The following morphometric traits were evaluated in fruits: Equatorial Diameter of the Fruit (EDF) in millimeters, measured using a digital caliper with a precision of 0.01 mm; Weight of 25 Fruits (WF) and Weight of 25 Seeds (WS) (g). The evaluations were conducted in a completely randomized design.

For seedling emergence analyses, the following traits were assessed: Emergence Speed Index (ESI), Mean Time to Emergence (MTE), and Emergence percentage (E) (%). Evaluations were conducted over 153 days in a greenhouse, using a completely randomized design with four replicates of 25 seeds per genotype. Seeds underwent dormancy breaking by soaking in water at 40 $^{\circ }\hbox {C}$ for 20 minutes^20^, and emergence was recorded every two days.

For seedling growth assessments, the following traits were measured: Shoot Dry Mass (SDM) (g), Total Dry Mass (TDM) (g), Dickson’s Quality Index (DQI)^21^, Leaf Area (LA) ($\hbox {cm}^{2}$), Diameter of Seedlings at the Base (DSB) (mm), and Seedling Height (SH) (cm). Evaluations followed a completely randomized design with five assessment time points over 262 days, with measurements taken from 10 replicates per genotype per evaluation. Seedlings were transplanted 153 days after sowing in sand (considered time zero) and acclimatized in a greenhouse under intermittent irrigation for 30 days.

From time zero onwards, destructive analyses were performed on 10 plants per genotype every 50 days, totaling five destructive assessments. On the last evaluation date, an additional 12 days of growth were included to enable chlorophyll fluorescence analysis, resulting in a total growth period of 262 days after transplanting.

Chlorophyll fluorescence was measured using the potential quantum efficiency of photosystem II ($\Phi _{PSII}$) ${F_v}/{F_m}$ with a FluorPen FP110 portable fluorometer (Photon Systems Instruments). Measurements were taken from the adaxial part of the largest leaf, in the intermediate region, avoiding necrotic spots and the main vein. Leaves were adapted to darkness for 30 minutes using metal clips before measurement, performed at 8:00 AM and noon, on a total of 248 plants.

EDF was measured from 10 replicates per genotype; WF and WS were evaluated in four replicates of 25 fruits; ESI was calculated according to Maguire’s equation^22^; MTE was calculated according to Labouriau and Viladares^23^; E was evaluated in four replicates of 25 seeds; SDM and TDM were assessed at five different time points with 10 replicates per genotype; DQI was calculated using the methodology of Dickson, Leaf, and Hosner^24^; SH and DSB were evaluated six times, including time zero, with 10 replicates per genotype.

### Genotyping by microsatellite markers

Genomic DNA from 59 each founder genotype was extracted from cortex samples using the protocol described by Carvalho^25^. DNA quality, concentration, and integrity were verified using a NanoDrop Thermo Scientific$\circledR$ spectrophotometer with a 260/280 absorbance ratio between 1.8 and 2.0, and through 0.8% agarose gel electrophoresis stained with GelRed (Biotium).

Thirteen SSR loci developed by Gaiotto, Brondani, and Grattapaglia^26^ for *E. edulis* were screened, out of which eight microsatellite markers generated reproducible banding pattern and scoring clarity to ensure the robustness of our dataset. Therefore, these eight markers (EE05, EE09, EE23, EE43, EE45, EE47, EE48 and EE52) were selected for final analysis. The polymerase chain reaction (PCR) was conducted in a final reaction volume of 20 $\upmu \hbox {L}$ following the manufacturer’s recommendations. Each reaction contained 30 ng of genomic DNA, 1X commercial Gotaq buffer, 0.3 $\upmu \hbox {M}$ of each primer (forward and reverse), 1.5 mM MgCl$_2$, 2.5 $\upmu \hbox {g}$ BSA, 0.25 mM dNTPs, and 1.25 U of Taq DNA polymerase (Promega). Amplifications were performed using a VeritiTM 96-Well Thermal Cycler (Thermo Fisher Scientific) with the following conditions: 2 cycles at 94 $^{\circ }\hbox {C}$ for 4 minutes, 35 cycles at 94 $^{\circ }\hbox {C}$ for 45 seconds, annealing at 55 $^{\circ }\hbox {C}$ for 1 minute, and extension at 72 $^{\circ }\hbox {C}$ for 1 minute, followed by a final extension at 72 $^{\circ }\hbox {C}$ for 10 minutes. PCR products were analyzed via 1.5% agarose gel electrophoresis stained with GelRed (Biotium).

PCR products were diluted based on their concentrations observed in agarose gel electrophoresis. For fragment analysis, 1.0 $\upmu \hbox {L}$ of each sample, 0.25 $\upmu \hbox {L}$ of $\hbox {GeneScan}^{\textrm{TM}}$ 600 $\hbox {LIZ}^{\textrm{TM}}$ Dye size standard (Thermo Fisher Scientific), and 8.75 $\upmu \hbox {L}$ of $\hbox {Hi-Di}^{\textrm{TM}}$ formamide (Thermo Fisher Scientific) were used. Samples were denatured at 95 $^{\circ }\hbox {C}$ for 3 minutes in a VeritiTM 96-Well Thermal Cycler and analyzed using a SeqStudio Genetic Analyzer (Thermo Fisher Scientific). Allele fragment sizes were determined using GeneMapper V5.0 software (Softgenetics, State College, PA, USA), considering peaks with relative fluorescence intensity above 350 RFU as valid. A table was compiled containing the allele sizes (bp) detected for each genotype for subsequent genetic diversity analyses.

### Data analysis

Estimates for quantifying genetic diversity via microsatellite molecular markers were obtained using the Software R^27^. The diversity of parameters addressed in the present work were the number of observed alleles ($N_{A}$), observed heterozygosity (Ho) (Eq. 1), expected heterozygosity (He)^28^ (Eq. 2), inbreeding coefficient (F) (Eq. 3) and the however polymorphic information measure (PIC)^29^ (Eq. 4). The estimators of the mentioned parameters are presented below:

1 $$\begin{aligned} H_o = \frac{N H_o}{N} \end{aligned}$$

where: $H _o$ is the frequency of observed heterozygotes or observed heterozygosity; $N H _o$ is the number of observed heterozygotes and *N* is the sample size.

The unbiased estimator of expected heterozygosity (H_e_) for genetic diversity within a population used in the present study was:

2 $$\begin{aligned} H_e = \frac{N}{N - 1} \left( 1 - \sum _{i=1}^{a} p_i^2 \right) \end{aligned}$$

where: $H_e$ is the unbiased estimator of expected heterozygosity; $p_i$ is the frequency of the i-th allele of the locus under study.

The inbreeding coefficient F can be estimated from the observed heterozygosity (Ho) and expected heterozygosity (H_e_) using the following equation:

3 $$\begin{aligned} F = 1 - \left( \frac{H_o}{H_e}\right) \end{aligned}$$

where: $F$ is the inbreeding coefficient.

The Polymorphic Information Content (PIC) is a measure of the informativeness of a genetic marker. It is calculated as:

4 $$\begin{aligned} PIC = 1 - \sum _{i=1}^{a} p_i^2 - \sum _{i,j=1, i \ne j}^{a} p_i^2 p_j^2 \end{aligned}$$

where: $p_j$ is the frequency of the j-th allele of the locus under study.

The estimates of variance components, genetic parameters, and genetic value predictions for the genotypes under study were performed individually for each trait using the restricted maximum likelihood method (REML) and the best linear unbiased prediction (BLUP)^30,31^. For all traits, the Likelihood Ratio Test (LRT) was conducted to evaluate the significance of the effects, ensuring that the final model included only significant effects (Table S1).

The adjusted model for biometric variables is given by:

5 $$\begin{aligned} \textbf{y} = {\textbf {1}}\mu + {\textbf {X}}_1 \textbf{b} + {\textbf {Z}}_1 \textbf{g} + \bf{\varvec{\varepsilon }}_1 \end{aligned}$$

where $\textbf{y}$ is the data vector ($n \times 1$), where *n* is the number of phenotypic observations; $\mu$ is a scalar corresponding to the model intercept; $\mathbf {X_1}$ is an incidence matrix of dimension $n \times r$, where *r* is the number of replications; $\textbf{b}$ represents the fixed effects vector with dimensions $r \times 1$; $\mathbf {Z_1}$ is an incidence matrix of dimension $n \times i$ associated with the vector of random effects of genetic $\textbf{g}$ with dimensions $i \times 1$, where *i* is the number of genotypes, $\textbf{g} \sim N(\textbf{0}, \sigma _g^2 \textbf{I}_i)$ were $\sigma _g^2$ is the genetic variance; and $\bf \varvec{\varepsilon _1}$ is the residual error vector with dimensions $n \times 1$, $\bf{\varvec{\varepsilon}_1} \sim N(\textbf{0}, \sigma _\varepsilon ^2 \textbf{I}_n)$, $\sigma _\varepsilon ^2$ is the residual variance. $\textbf{I}_x$ is an identity matrix with a dimension determined by its subscript [x = *i* is the number of genotypes; *n* is the number of phenotypic observations]. The full model initially tested included the fixed effect of field fruit samplings and the random effect of sampling elevation. However, both effects were not significant based on the corrected AIC and BIC criteria for fixed effects^32^, as well as the AIC and BIC tests, respectively.

For emergence variables, the adjusted model includes an additional random effect:

6 $$\begin{aligned} \textbf{y} = {\textbf {1}}\mu + {\textbf {X}}_2 \textbf{b} + {\textbf {Z}}_1 \textbf{g} + {\textbf {Z}}_2 \textbf{al} + \bf{\varvec{\varepsilon}_2} \end{aligned}$$

where $\mathbf {X_2}$ is an incidence matrix of dimension $n \times m$, where *m* is the number of replications (*r*) added to the number of field fruit samplings (*c*); $\textbf{b}$ represents the fixed effects vector with dimensions $m \times 1$; $\mathbf {Z_2}$ is an incidence matrix of dimension $n \times l$ associated with the vector of random effects of sampling elevation $\textbf{al}$ with dimensions $l \times 1$, where *l* is the number of sampling elevation, $\textbf{al} \sim N(\textbf{0}, \sigma _{al}^2 \textbf{I}_l)$ where $\sigma _{al}^2$ is the sampling elevation variance; $\varvec{\varepsilon _2} \sim N(\textbf{0}, \textbf{R} \otimes \textbf{I}_n)$, $\sigma _\varepsilon ^2$ is the residual variance and R is the variance-covariance (VCOV) residual matrix for the field fruit samplings with dimensions $c \times c$, where *c* is the number of field fruit samplings; $\otimes$ is the Kronecker product.

The model for seedling growth variables considers genotype-by-evaluation interaction:

7 $$\begin{aligned} \textbf{y} = {\textbf {1}}\mu + {\textbf {X}}_3 \textbf{b} + {\textbf {Z}}_1 \textbf{g} + {\textbf {Z}}_3 \textbf{gm} + \bf{\varvec{\varepsilon}_3} \end{aligned}$$

where $\mathbf {X_3}$ is an incidence matrix of dimension $n \times o$, where *o* is the number of replications (*r*) added to the number of different evaluations times (*t*); $\textbf{b}$ represents the fixed effects vector with dimensions $o \times 1$; $\textbf{gm}$ is the $it\times 1$ vector of random effects genotype-by-evaluations, with $\textbf{gm} \sim N(\textbf{0}, \textbf{G}_{gm} \otimes \textbf{I}_i)$; The matrix ${\textbf {Z}}_3$ is the incidence matrix for random effect *gm*; $\boldsymbol{\varvec{\varepsilon _3}} \sim N(\textbf{0}, \textbf{R} \otimes \textbf{I}_n)$, $\sigma _\varepsilon ^2$ is the residual variance. $\boldsymbol{G}_{gm}$ is the VCOV matrix for the effect of genotypes between evaluations with dimensions $t \times t$; *R* is the residual VCOV.

For the physiological variable group ($F_{v}/F_{m}$), the adjusted model simplifies to:

8 $$\begin{aligned} \textbf{y} = {\textbf {1}}\mu + {\textbf {X}}_4 \textbf{b} + {\textbf {Z}}_1 \textbf{g} + \bf{\varvec{\varepsilon}_4} \end{aligned}$$

where $\mathbf {X_4}$ is an incidence matrix of dimension $n \times k$, where *k* is the number of replications (*r*) added to the number of different evaluations times of the $F_{v}/F_{m}$ (*h*); $\textbf{b}$ represents the fixed effects vector with dimensions $k \times 1$; $\bf{\varvec{\varepsilon}_4} \sim N(\textbf{0}, \textbf{R} \otimes \textbf{I}_n)$.

The significance of the effects tested in these models was assessed using LRT, and the corrected Bayesian Information Criterion (BICc)^33^ was used to determine the inclusion of collection effects, evaluation times, and measurement times as fixed effects (Table S1).

To improve genetic value predictions, different assumptions were applied for $\textbf{G} (i \times i)$ and $\textbf{R} (n \times n)$, using various covariance structures^34–37^. The selection of the best model was performed using the Akaike^38^ and Bayesian^39^ information criteria (Table S2).

The broad-sense heritability $H^2$ was estimated as^40^:

9 $$\begin{aligned} H_g^2 = 1 - \frac{\overline{\Delta }_{BLUP}}{2\sigma _g^2} \end{aligned}$$

where: $\overline{\Delta }_{BLUP}$ represents the average prediction error variance between genotype pairs, and $\sigma _g^2$ is the genotypic variance.

Prediction accuracy $r$ was estimated as:

10 $$\begin{aligned} r = \sqrt{1 - \frac{PEV}{\sigma _g^2}} \end{aligned}$$

where: $PEV$ denotes the prediction error variance.

To evaluate the variability among genotypes, the LRT compared the reduced and full models, with significance assessed using the chi-square ($\chi ^2$) test^41^. Correlation estimates were computed as:

11 $$\begin{aligned} r_{y_1 y_2} = \frac{\text {Cov}(y_1, y_2)}{\sqrt{\sigma _{y_1}^2 \times \sigma _{y_2}^2}} \end{aligned}$$

where $\text {Cov}(y_1, y_2)$ denotes the covariance between trait pairs, and $\sigma _{y_1}^2$ and $\sigma _{y_2}^2$ represent the variances of the respective traits.

Before conducting path analysis, the Variance Inflation Factor (VIF) method of Montgomery and Peck^42^ was applied to test for multicollinearity, and traits with $VIF> 5$ were excluded from the analysis. The analysis was based on the phenotypic correlation matrix, considering the main effect characteristic, DQI.

For genetic diversity analysis, only genotypes included in both phenotypic and molecular evaluations (59 founder genotypes) were considered. The analysis consisted of two stages: first, clusters formed by BLUP-predicted genotypic data and microsatellite molecular data were compared using standardized mean Euclidean distance (DEMP) for genotypic data and the unweighted index for molecular data^43^. In the second stage, dissimilarity matrices were normalized to a scale from zero to one, and an average matrix of both normalized matrices was constructed. The UPGMA method was employed for clustering, and the number of groups was determined using Mojema’s criterion^44^ with $k = 1.25$. The association significance between matrices was tested using the Mantel test^45^ with 5000 permutations. All statistical analyses were conducted in R^46^.

## Results

### Phenotypic data

**Fig. 2:**
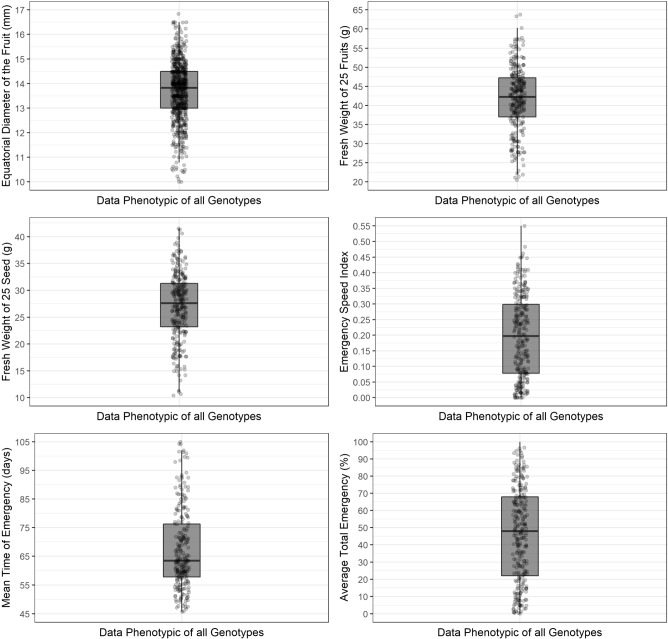
Figures describing the phenotypic data of all measurements performed on all founders genotypes sampled for the traits equatorial diameter fruit (EDF) (mm); weight of 25 fruits (WF) (g); weight of 25 seeds (WS) (g); emergency speed index (IVE); mean time to emergence (MTE) (days) and emergency (E) (%).

**Fig. 3:**
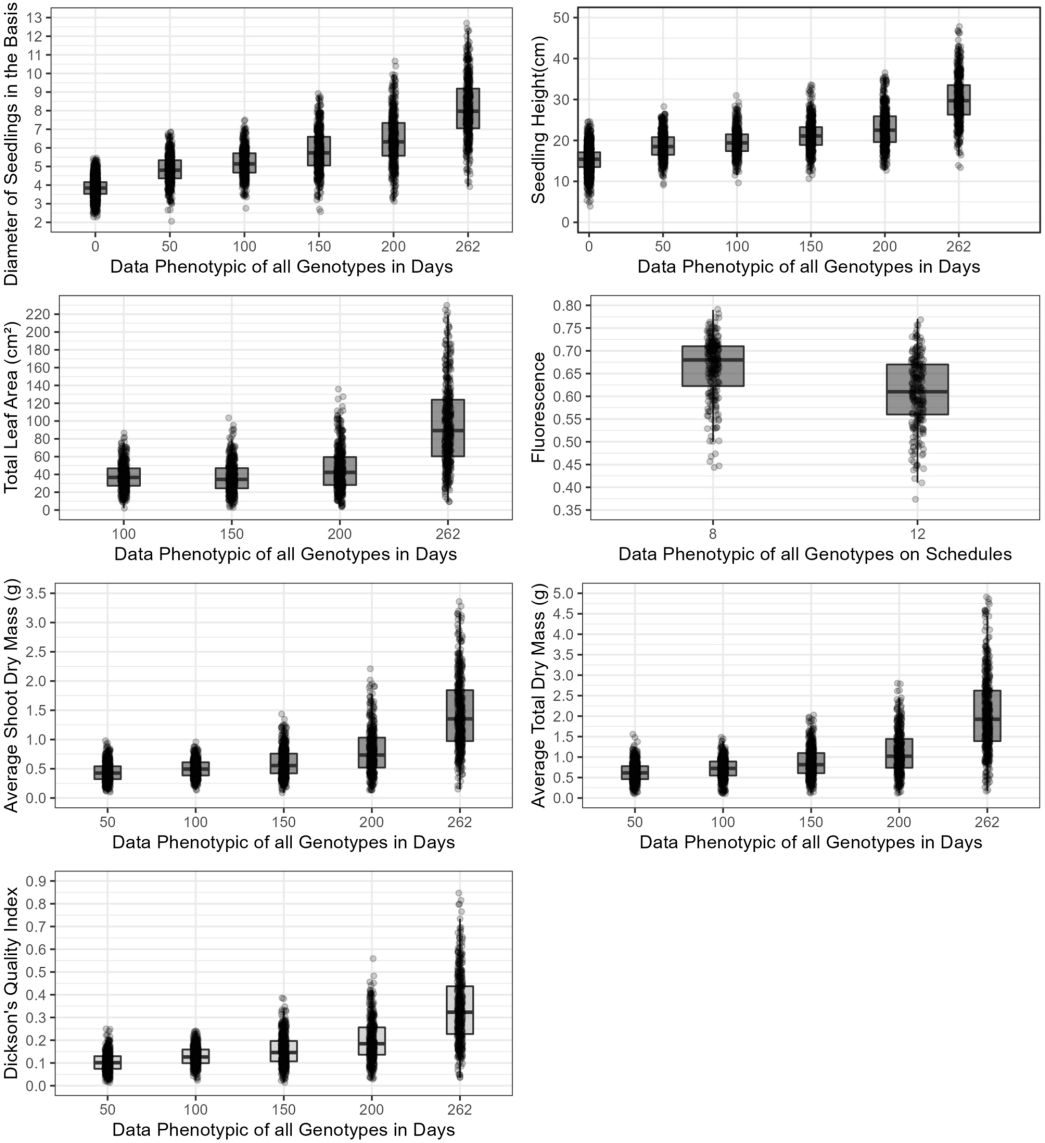
Figures describing the phenotypic data of all measurements performed on all founders genotypes sampled for the traits diameter to collar height (DSB) (cm); seedling height (SH) (cm); leaf area (LA) ($\hbox {cm}^{2}$) and chlorophyll fluorescence ($F_{v}/F_{m}$), shoot dry mass (SDM) (g); total dry mass (TDM) (g), Dickson quality index (DQI). The $\times$ axis is defined for measurement days (DSB, SH, LA, $F_{v}/F_{m}$, SDM, TDM, DQI) and measurement time ($F_{v}/F_{m}$).

Substantial variation in phenotypic traits was observed between the founders genotypes, sampled to compose the breeding foundation population, as indicated by the descriptive analysis of the phenotypic data, graphical boxplot analysis, and data distribution, including means for the 13 evaluated traits (Fig. 2 and Fig. 3).

### Variance components and genetic parameters

Genetic variability was detected between genotypes for all evaluated traits, as evidenced by the significance of the maximum likelihood ratio test (LRT) using the $\chi ^2$ test. These results, along with the estimates of variance components and genetic parameters, are presented in Table 1.

**Table 1:** Estimates of variance components and genetic parameters in a breeding foundation population of *E. edulis* for: equatorial diameter fruit (EDF) (mm), weight of 25 fruits (WF) and seed (WS) (g), emergence speed index (ESI), mean emergence time (MET), percentage of emergence (E), shoot dry mass (SDM) (g), total dry mass (TDM) (g), Dickson’s quality index (DQI), leaf area (LA) ($\hbox {cm}^{2}$), diameter at stem height (DSB) (mm), seedling height (SH) (cm) and variable fluorescence/maximal fluorescence ($F_{v}/F_{m}$).

Group	Traits	$H^2$	*r*	AIC	BIC	LRT1	LRT2
Biometric	EDF	0.98	0.99	89.60	98.74	909.7***	–
	WF	0.99	0.99	727.41	734.72	847.39***	–
	WS	0.99	0.99	641.22	648.54	761.91***	–
Emergence	ESI	0.29	0.72	-1079.09	-1061.21	237.05***	0.12ns
	MET	0.76	0.87	1423.06	1437.23	173.91***	–
	E	0.69	0.83	1424.76	1442.47	224.66***	$1.01\times 10^{-5}$ ns
Seedling Growth	DSB	0.87	0.93	326.23	409.40	383.41***	997.38***
	SH	0.90	0.95	13210.19	13267.65	561.34***	718.48***
	LA	0.85	0.92	12450.09	12499.53	160.71***	227.04***
	SDM	0.81	0.90	-7133.73	-7070.29	158.12***	1561.1***
	TDM	0.83	0.91	-3081.11	-3017.20	166.86***	261.73***
	DQI	0.84	0.91	-10636.38	-10573.81	190.34***	425.63***
Physiological	$F_{v}/F_{m}$	0.71	0.84	-2072.45	-2059.94	37.34***	$-3.86\times 10^{-5}$ ns

LRT1: Maximum likelihood test for the variance component $\sigma _g^2$. LRT2: Maximum likelihood test for interaction effects. The tested effects were: Emergence traits: $\sigma _{gal}^2$ (variance of genotype $\times$ elevation interaction). Seedling growth traits: $\sigma _{gm}^2$ (variance of genotype $\times$ measurement interaction). Physiological traits: $\sigma _{gh}^2$ (variance of genotype $\times$ time interaction). *** Significant based on a $\chi ^2$ test with 1 degree of freedom at the 1% probability level.

For biometric traits (EDF, WF, and WS) and emergence traits (ESI, MTE, and E), genetic effects had a greater influence on phenotypic expression, as indicated by higher estimates of $\sigma _g^2$ compared to $\sigma _e^2$ (Table 1). For seedling growth traits (SDM, TDM, DQI, LA, DSB, SH, and $F_{v}/F_{m}$), residual effects had a more pronounced impact on phenotypic expression. Consequently, heritability ($H^2$) values were higher for biometric and emergence traits (ranging from 0.75 for MTE to 0.99 for WF) compared to seedling growth traits (ranging from 0.22 for TDM to 0.32 for LA).

### Genetic and phenotypic correlations

**Fig. 4:**
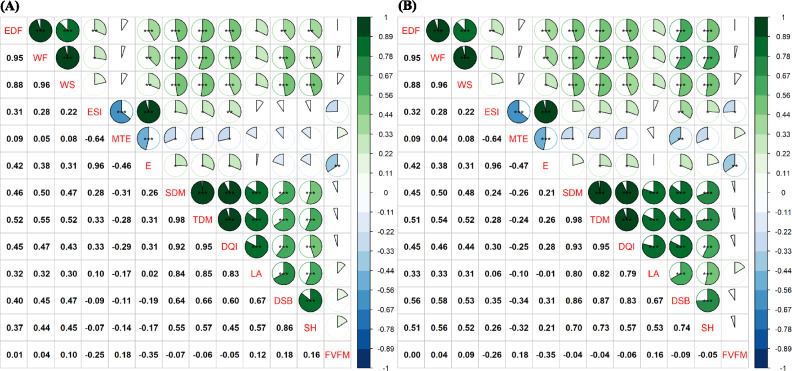
Phenotypic (rf) (**A**) and genetic (rg) (**B**) correlation between traits. On the upper diagonal, the significance and correlation are presented graphically, while on the lower diagonal, the correlation is presented numerically for the characteristics, namely: equatorial diameter of the fruit (EDF) (mm), weight of 25 frutis (WF) and seed (WS) (g), emergence speed index (ESI), mean emergence time (MTE), percentage of emergence (E), shoot dry mass (SDM) (g), total dry mass (TDM) ( g), Dickson quality index (DQI), total leaf area (LA) ($\hbox {cm}^{2}$), diameter of seedling in the basis (DSB) (mm), seedlings height (SH) (cm) and variable fluorescence/maximum fluorescence ($F_{v}/F_{m}$).

Most correlation estimates among the 13 evaluated traits were significant at 1%. In general, genetic correlations ($r_g$) were higher than phenotypic correlations ($r_f$) (Fig. 4A and Fig. 4B). Fifty-three significant phenotypic correlations were identified, with coefficients ranging from -0.64 to 0.96. Among these, 35 were significant at 0.1%, 7 at 1%, 12 at 5%, and 24 were not significant (Fig. 4A). Among genetic correlations, 55 were significant, with coefficients ranging from -0.64 to 0.96, including 37 at 0.1%, 5 at 1%, 14 at 5%, and 22 not significant (Fig. 4B).

The genetic correlations between fruit biometric traits (EDF, WF, and WS) were all significant and above 0.88, indicating a strong association. For emergence traits (ESI, MTE, and E), both positive and negative correlations were observed, with the highest correlation occurring between ESI and E (0.96) (Fig. 4B), suggesting a strong linear association between early-emerging seedlings and higher emergence rates. MTE exhibited several negative associations, particularly with E (-0.47). The genetic correlations between fruit biometric traits (EDF, WF, and WS) and dry mass seedling traits (SDM and TDM) exhibited moderate association values, ranging from 0.45 to 0.54 (Fig. 4). SDM and TDM showed stronger associations with DSB (0.86 and 0.87, respectively) and SH (0.70 and 0.73, respectively).

The mean genetic correlation ($r_g$) between the DQI and biometric traits of fruit and seed (EDF, WF, and WS) ranged from 0.44 to 0.46, indicating a moderate association between fruit/seed size and seedling quality. Among emergence traits, DQI was significantly correlated only with E (0.28), demonstrating a clear linear association. For growth traits, there was a strong positive correlation between DSB and SH (0.74), showing that seedling height growth follows diameter growth. The physiological parameter $F_{v}/F_{m}$ showed no significant linear associations with most traits, except for E (-0.35).

### Path analysis

**Fig. 5:**
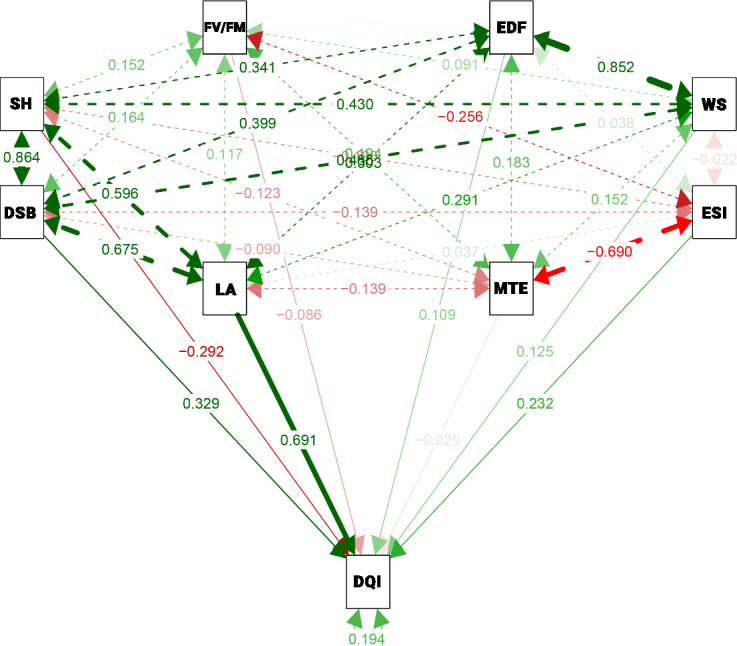
Path analysis between the characteristics equatorial diameter of the fruits (EDF) (mm), eight of 25 seeds (WS), emergence speed index (ESI), mean emergence time (MTE), Dickson’s quality index (DQI), total leaf area (LA) ($\hbox {cm}^{2}$), diameter of seedlings in the basis (DSB) (mm), seedlings height (SH) (cm) and variable fluorescence/maximal fluorescence ($F_{v}/F_{m}$). Coefficient of determination ($\hbox {R}^{2}$) = 80.57%; residual variable effect (EVR) = 0.4408.

Correlation analysis alone can lead to misinterpretations due to indirect effects among explanatory traits. Path analysis was performed to partition the estimates of phenotypic correlations into direct and indirect effects. The analysis is presented in Fig. 5, illustrating the direct effects of explanatory variables on DQI and their correlations (top).

The multicollinearity study was conducted, and traits WF, ESI, MTE, and E were removed to mitigate variance inflation factors. The coefficient of determination ($R^2$) was 80.57%, with a residual effect (EVR) of 0.44, indicating a good model fit for explaining DQI variations. Of the remaining explanatory traits, only $F_{v}/F_{m}$ had a negative direct effect (-0.09) greater than the phenotypic correlation coefficient (-0.06) (Fig. 4B). SH, $F_{v}/F_{m}$, and MTE had direct negative effects (-0.29, -0.09, and -0.02, respectively) on DQI. LA (0.69) and DSB (0.33) (Fig. 4) had the most substantial direct influence on DQI, with LA standing out due to its high magnitude exceeding the residual effect (EVR = 0.44).

### Microsatellite marker analysis

We initially tested a total of 13 microsatellite markers, only eight yielded consistent and reliable amplification across all individuals analyzed. In this eight evaluated loci, a total of 118 alleles were identified. The number of alleles per locus ranged from 8 (EE43) to 20 (EE05), with an average number of alleles per locus ($N_m$) of 14.75. All evaluated loci exhibited high polymorphic information content (PIC $\ge$ 0.5). Observed heterozygosity ($H_O$) values ranged from 0.37 (EE09) to 0.98 (EE52), while expected heterozygosity ($H_e$) varied between 0.73 (EE43) and 0.93 (EE05). Inbreeding coefficient (*F*) estimates ranged from -0.13 (EE45) to 0.52 (EE09). Most loci showed negative *F* values, except for EE09 (0.52) and EE43 (0.04), which had positive values (Table 2). All other estimates should be considered null since their values were negative. As additional information for population diversity characterization, the mean PIC values were estimated, ranging from 0.68 (EE43) to 0.92 (EE05 and EE47), with an average of 0.84 (Table 2).

**Table 2:** Genetic diversity indices derived from the analysis of eight microsatellite loci in founder genotypes sampled in the state of Espírito Santo, Brazil, for the establishment of the breeding foundation population and conservation of *E. edulis*.

Locus	*N*	$N_A$	$H_o$	$H_e$	*F*	PIC
EE05	58	20	0.95	0.93	$-$0.02	0.92
EE09	49	9	0.37	0.77	0.52	0.74
EE23	55	14	0.93	0.87	$-$0.07	0.85
EE43	53	8	0.70	0.73	0.04	0.68
EE45	59	14	0.93	0.83	$-$0.13	0.81
EE47	58	19	0.95	0.92	$-$0.03	0.92
EE48	58	17	0.95	0.88	$-$0.07	0.87
EE52	53	17	0.98	0.92	$-$0.07	0.91

*N*: sample size; $N_A$: number of observed alleles; $H_o$: observed heterozygosity; $H_e$: expected heterozygosity; *F*: inbreeding coefficient.

### Diversity analysis

**Fig. 6:**
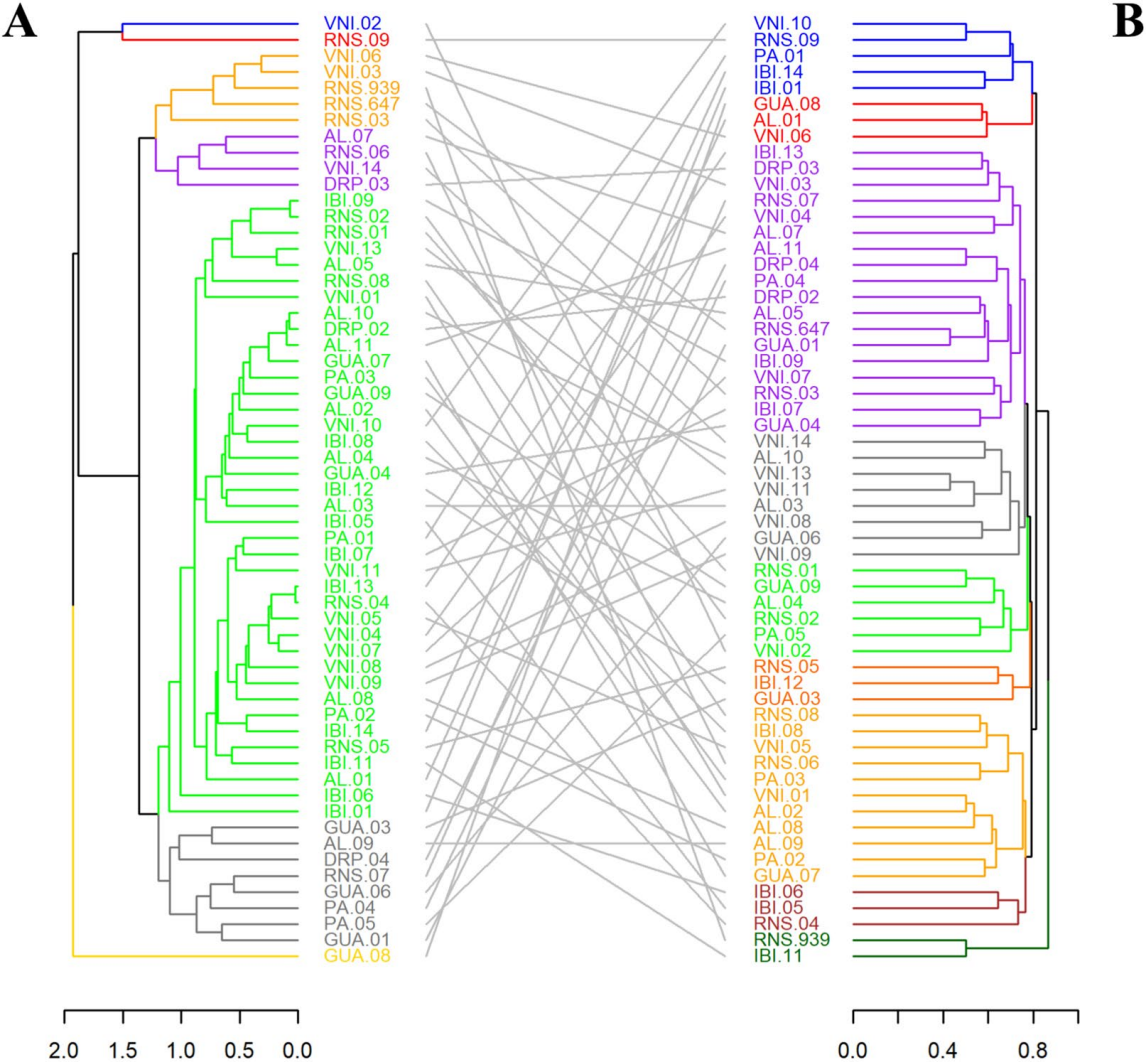
Dendrograms generated based on dissimilarity values, by the predicted BLUP’s for each founders genotypes (genetic value) (**A**) and by molecular markers (**B**), obtained by the Standardized Mean Euclidean Distance (DEMP) and by the unweighted index (INP), respectively. Both clusters are generated by the method of average linkage between groups (UPGMA) and cut-off point with k=1.25. The different colors represent each considered group. The initial acronyms refer to the municipalities where the founders genotypes was sampled, being: Alegre (AL), Domingos Martins (PA), Dores do Rio Preto (DRP), Guaçuí (GUA), Ibitirama (IBI), Rio Novo do Sul (RNS) and Venda Nova do Imigrante (VNI) Groups formed in A: Group I (blue), Group II (red), Group III (orange), Group IV (purple), Group V (green), Group VI (gray) and Group VII (yellow). Groups formed in B: Group I (blue), Group II (red), Group III (purple), Group IV (grey), Group V (green), Group VI (orange), Group VII (yellow), Group VIII (brown) and Group IX (dark green).

Genetic and molecular traits were used to assess genetic divergence. Genetic data were derived from BLUP estimates obtained via REML/BLUP. The greatest observed genetic distance (3.07) was between RNS09 and VNI14, while the smallest (0.02) was between IBI13 and RNS04, with a mean distance of 1.15. Seven groups were identified (Fig. 6A).

Microsatellite analysis showed genetic differences among genotypes from both geographically proximate (GUA.06 and AL.02) and distant populations (GUA.08 and PA.01). The smallest genetic distance (0.4285) was observed between RNS647 and GUA01. Nine groups were identified (Fig. 6B), with genetic distances ranging from 0.42 to 1.00 and a mean of 0.78.

**Fig. 7:**
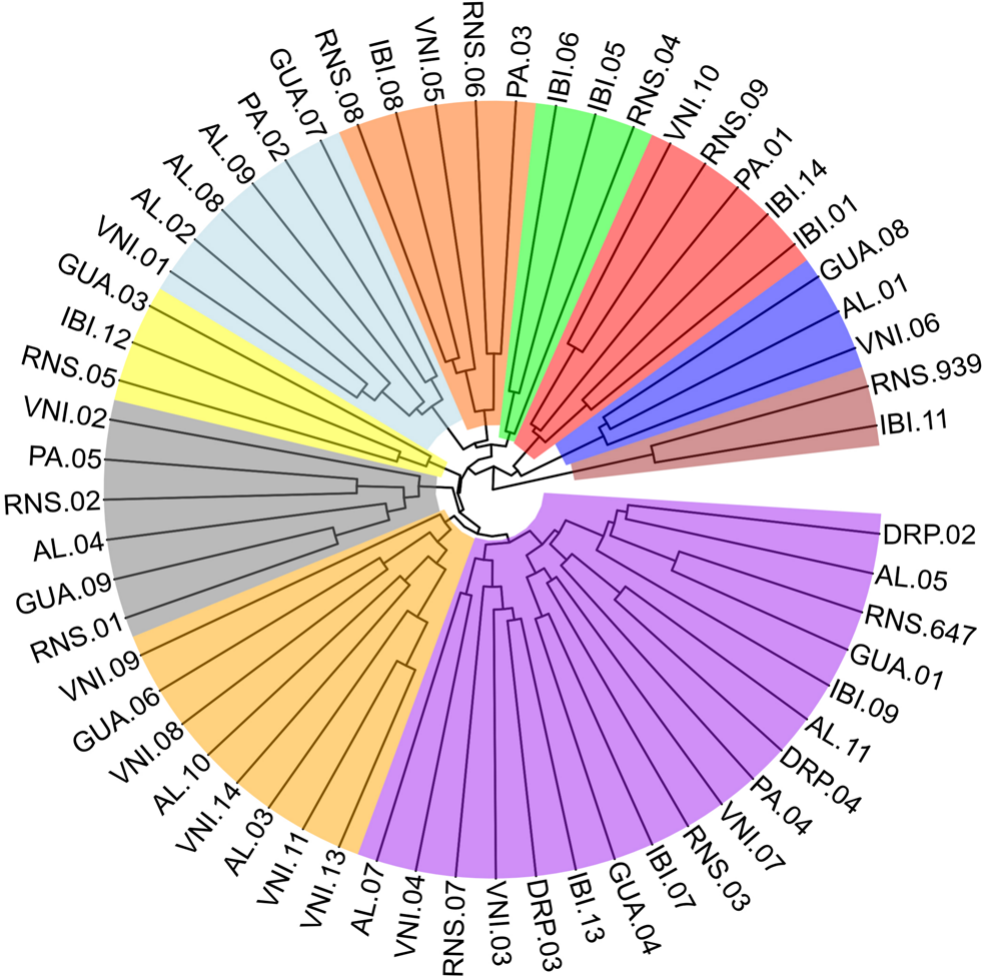
Dendrograms generated based on the average dissimilarity values of the individual genotypic and molecular distance matrices, obtained by the Standardized Mean Euclidean Distance method (DEMP) and by the unweighted index (INP), respectively. The clustering was generated by the method of mean linkage between groups (UPGMA) and the cut-off point determined according to Mojema^44^ with k=1.25. The different colors represent each considered group. The initial acronyms refer to the municipalities where the matrix was sampled, being: Alegre (AL), Domingos Martins (PA), Dores do Rio Preto (DRP), Guaçuí (GUA), Ibitirama (IBI), Rio Novo do Sul (RNS) and Venda Nova do Imigrante (VNI). Groups formed: Group I (purple), Group II (orange), Group III (gray), Group IV (yellow), Group V (light blue), Group VI (dark orange), Group VII (green), Group VIII (red), Group IX (dark blue) and Group X (brown).

A Mantel test revealed no correlation between the genetic and molecular distance matrices (r = 0.098, p-value = 0.022). The combined analysis resulted in a mean genetic distance of 0.49, with a minimum of 0.10 (VNI11 and VNI13) and a maximum of 0.89 (IBI06 and VNI02). Ten distinct groups were identified based on joint analysis (Fig. 7).

## Discussion

### Genetic analysis

The results of this study indicate genetic variability among genotypes for all evaluated traits, with biometric (EDF, WF, WS) and emergence traits (ESI, MTE, E) showing strong genetic control, as demonstrated by their high heritability values (Table 1). This is particularly relevant for breeding programs, as high heritability facilitates the selection process and enhances the potential for genetic gain across generations. In addition, traits such as fruit and seed size (WF and WS) and emergence performance (e.g., ESI) not only exhibited high heritability but also showed positive genetic correlations with seedling quality (e.g., DQI). This suggests that selecting genotypes with desirable fruit and seed traits may indirectly enhance seedling vigor - a key objective in both conservation and domestication efforts for *E. edulis*. On the other hand, traits related to seedling growth appear to be more influenced by environmental factors, emphasizing the need for robust experimental designs and evaluations under diverse conditions to increase selection accuracy^47^. In this context, the observed results are crucial for guiding the development of a genetic improvement program for *E. edulis*. They enhance the understanding of the genetic control of the evaluated traits and their associations, enabling breeders to design more effective strategies across experimental, analytical, and selection processes, ultimately aiming to maximize genetic gains.

Morphophysiological growth traits-such as seedling height (SH), leaf area (LA), shoot and total dry mass (SDM, TDM), and chlorophyll fluorescence ($F_{v}/F_{m}$) - generally exhibited lower heritability estimates in our study due to a greater influence of environmental variation and genotype $\times$ environment interactions over time. These traits are measured during the seedling developmental phase and are highly plastic, being affected by subtle variations in microenvironmental conditions (e.g., light, temperature, humidity, soil heterogeneity); additionally, these traits often involve complex physiological processes that are controlled by multiple genes with small effects, making the genetic contribution more diffuse. As a result, the residual variance ($\sigma _e^2$) tends to be higher relative to the genetic variance ($\sigma _g^2$), leading to lower heritability estimates ($H^2$). In contrast, fruit and seed traits are typically less plastic and more developmentally stable, especially when measured at a single time point, which contributes to higher heritability values. These results for fruit-related traits are consistent with previous studies^11,48,49^, which have also reported elevated values in multi-year evaluations^50^. These findings reinforce the strong genetic control of these morpho-agronomics traits, which holds considerable relevance for the fruit processing industry. In summary, the lower heritability estimates for morphophysiological traits reflect their greater environmental sensitive.

Given their relevance as variable, informative, and robust markers^51^, capable of detecting high levels of polymorphism^52^ and effectively identifying genetic divergence among genotypes^53,54^, we deliberately employed Simple Sequence Repeat (SSR) molecular markers in this study. This choice aimed to assess the molecular genetic diversity within the sampled population for the construction of its breeding foundation population. This strategic approach was adopted to validate the efficiency of the sampling process, providing a comprehensive analysis of genetic diversity, as well as allele identification and distribution. These findings are crucial to ensuring the success of the *E. edulis* conservation and breeding program, offering fundamental insights to enhance the efficiency and sustainability of the initiative. Although the number of markers used was limited (eigth), they were sufficiently informative and successfully discriminated against genotypes into distinct genetic groups. These markers used here have been validated in previous studies. For example, Pereira et al.^55^ employed nine microsatellite markers, including seven of those used in our study, to investigate *E. edulis* populations from different regions of Brazil. Their findings corroborate our results, reinforcing the patterns of genetic structure and diversity we report here. Additionally, previous analyses comparing high-throughput SNP markers and eigth SSR markers have confirmed similar patterns of genetic differentiation and diversity^54^, supporting the conclusions derived from our microsatellite data.

The results presented in Table 2 indicate that all analyzed loci can be classified as highly polymorphic and effective for analysis since the observed heterozygosity ($H_O$) values were above 0.70. The term polymorphism refers to the presence of multiple variations of a specific gene^56^. In genetic diversity studies, highly polymorphic loci are crucial as they reflect strong genetic variation that can be exploited for precise differentiation of genetic materials^57–59^.

Similar to $H_O$, the polymorphic information content (PIC) is an informativity estimate used to infer the degree of polymorphism of a given marker^58^. PIC estimates are classified as poorly informative when below 0.25, moderately informative between 0.25 and 0.50, and highly informative when exceeding 0.50^29^. Accordingly, all markers used in this study can be classified as highly informative, as the lowest estimated PIC value was 0.68 for locus EE43.

The observed *F* values for most loci were negative, indicating heterozygote excess and confirming allele exchange among genotypes within populations. Positive *F* values were found for loci EE09 and EE43, suggesting an excess of homozygotes. Since *E. edulis* is predominantly allogamous, an acceptable self-fertilization rate (5%) may explain the increased homozygosity at these loci.

The results of this study support the effectiveness of sampling broad genetic variability to establish a breeding fundation population or a germplasm collection for species conservation. This effectiveness is particularly evident when compared to previous studies, which, although indicating high diversity, presented results similar to or lower than those observed in this study Carvalho et al.^60^($H_e$: 0.78-0.86); Coelho et al.^61^ (N: 250; $H_e$: 0.61-0.75; *F*: 0.05-0.36); Conte et al.^62^ (N: 600; $H_e$: 0.781-0.785); Carvalho et al.^63^ (N: 463; $H_e$: 0.72-0.86; *F*: 0.10-0.29); Gaiotto et al.^64^(N: 583; $H_e$: 0.73-0.79; *F*: low or null); Novello et al.^65^ (N: 361; $H_e$: 0.76-0.83; *F*: 0.10-0.34); Pereira et al.^55^ (N: 527; $H_e$: 0.54-0.81; *F*: 0.03-0.28).

Genetic diversity is a key factor in the construction of germplasm banks and the development of founder populations, not only for species conservation but also for providing a diverse set of characteristics that can be explored in breeding programs. The markers used identified a total of 118 alleles in 59 genotypes. This extensive allelic sampling becomes even more evident when compared to other studies, such as the genetic diversity characterization of *E. edulis* conducted by Pereira et al.^55^. In their study, the authors used nine SSR markers to evaluate 527 individuals from 26 locations along the species’ natural distribution in Brazil, identifying a total of 178 alleles.

Moreover, this study effectively demonstrated the high genetic variability of *E. edulis* in Espírito Santo, surpassing previous studies with lower diversity estimates. Specifically, Carvalho et al.^66^ assessed 160 genotypes in four populations using seven SSR markers, reporting $H_O$: 0.36-0.68, $H_e$: 0.60-0.79, $N_{A}$: 43, and *F*: 0.14-0.34. Additionally, Pereira et al.^55^ reported values for the same region: $H_O$: 0.40-0.60, $H_e$: 0.80, and *F*: 0.10-0.30. Thus, this study significantly contributes to the understanding and conservation of the species’ genetic diversity in the region.

Previous studies indicate that most of the genetic variation in *E. edulis* is concentrated within populations^55,62,66^. While confirming this diversity pattern, other researchers emphasize that population differentiation, although low, is statistically significant^64,65^. Interpreting these findings with caution is essential, as misinterpretation could lead to inadequate decision-making, particularly in promoting genetic material collection concentrated in only a few locations. This can be illustrated by drawing a parallel with the study conducted by Carvalho et al.^66^, which, although it provided a good estimate of genetic diversity (with an average of approximately 6 alleles per marker), may underestimate the genetic variation among populations. In contrast, the approach adopted in the present study, based on a broader sampling strategy that prioritizes the inclusion of genotypes from a larger number of populations across diverse geographic locations, proved to be more effective. This strategy resulted in a higher average number of alleles per marker (approximately 15), offering greater efficiency for conservation programs and a broader genetic base to be explored in breeding initiatives. This is particularly advantageous because the number of sampled alleles directly reflects the genetic diversity of a germplasm collection or breeding base population, which is the driving force behind selection and population evolution, for favoring resistance to pests and diseases, enhances adaptation to a wide range of biotic and abiotic stresses, and supports improvements in quality and yield of the cultures^67^.

This study underscores the importance of a broader genetic sampling approach for *E. edulis*, covering a large number of geographically distant populations. This strategy has proven effective in maximizing the species’ genetic diversity, aligning with conservation objectives. Accordingly, a sampling approach that maximizes the number of collection sites with a reduced number of genotypes per population (considering the effective population size, forest fragment size, and a minimum distance of 150 meters between genotypes) may be an efficient strategy for constructing a high-quality germplasm collection and breeding foundation population for *E. edulis*.

### Path analysis

Only the analysis of correlation coefficients can provide misinterpretations between the character associations^68^, since the estimates have indirect effects of other variables, so that the correlation estimates do not make it possible to verify the causes of direct effects^69^. Thus, it is necessary to carry out path analysis, as it allows the decomposition of correlation estimates into their direct and indirect effects, allowing the knowledge of the influences of the explanatory characters on the one of greatest interest, complementing the results obtained by the correlations and making it possible to verify the real causes and effects between the characteristics^69^.

With the removal of traits exhibiting VIF values greater than five, the path analysis yielded an $R^2$ estimate exceeding 0.70 and an EVR of 0.4408, indicating a good model fit for explaining variations in IQD. The presence of at least one trait with a VIF greater than five is sufficient for the associated regression coefficients to be highly influenced by multicollinearity^44^, highlighting the importance of excluding such traits from the analysis. In cases of high correlation but low direct effect, the most effective strategy for achieving satisfactory improvements in the primary variable is the simultaneous selection of traits, with emphasis on those exhibiting significant indirect effects^43^.

Among the evaluated traits, seedling height (SH) was the most notable due to the magnitude of its alteration and the shift in sign between the associations observed in the phenotypic correlation analysis (0.45) and in the path analysis (-0.29). The magnitude of this change, along with the shift in the direction of the association, underscores the need for caution when selecting seedlings based solely on shoot height (SH). By decomposing the direct effect of SH on DQI through path analysis (Fig. 5), it was observed that the associations identified in Fig. 4 were strongly influenced by the indirect effects of DSB and LA, since plants with higher expression of these traits tend to exhibit greater SH. Therefore, when isolating the direct effect of SH on DQI, path analysis may have revealed potential effects such as seedling etiolation responses, which can lead to lower-quality genotypes, i.e., reduced DQI. Depending on the light intensity, seedlings may exhibit an etiolated growth pattern^70^, which is finely regulated by process controlled by hormones, primarily auxins (IAA) and gibberellins (GAs), which promote cell elongation^71^. Excessive stem elongation has been widely recognized since the discovery of the first gibberellin (GA), which was found to cause lodging and yield loss in rice^72^. The genes GA 20-oxidase (GA20ox) and GA 2-oxidase (GA2ox) encode, respectivelly, enzymes that catalyze the biosynthesis and inactivation of gibberellins, regulated the accumulation of active GAs^71^, crucial for normal stem development and seedling quality.

However, in *E. edulis*, a climax species^73^ that requires shading during its early developmental stages^74,75^-a condition provided in the present experiment through 50% shade-we hypothesize that reduced light availability may have led to lower levels of the far-red absorbing form of phytochrome (Pfr). This, in turn, could increase tissue sensitivity to gibberellins (GAs) and consequently upregulate the expression of *GA20ox*, as previously observed in pea (*Pisum sativum* L.)^76^. This mechanism may have contributed to the observed elongation (etiolation) of *E. edulis* seedlings. Nevertheless, under such environmental conditions, apical meristematic cells may be deprived of metabolic sugars that directly regulate the expression of *CYCB1;1/CDKB1;1*, along with a decline in *CYCD3;1* activity, which is essential for the activation of cell division in meristematic regions^77^.

Thus, the isolated use of SH for selecting higher-quality seedlings should be avoided, as its direct effect is inversely proportional to DQI, potentially leading to undesirable outcomes. However, when considering SH for selecting seedlings with higher DQI, leaf area (LA) and dry stem biomass (DSB) should be used simultaneously, as they were the primary traits explaining variations in DQI, and presents high indirect effect level on SH. In a study on *Syagrus romanzoffiana* (Cham.), a positive correlation between DSB and DQI (0.75) was also observed, confirming the effectiveness of DSB assessment in indicating seedling quality^78^. These findings indicate that, for identifying seedling quality in visual assessments or future analyses, DSB and LA stand out as the most promising traits due to their ease of measurement and strong direct effect on DQI. Therefore, Pearson correlation was valuable for identifying associations among fruit, seed, and seedling traits, suggesting that larger fruits and seeds are generally linked to more vigorous seedlings. However, to clarify whether these associations were direct or indirect by other traits, we conducted path analysis using DQI as the response variable. This approach revealed that the influence of fruit and seed traits on seedling quality is mostly indirect, acting through traits such as leaf area and stem diameter.

### Diversity analysis

The observed genotypic divergence values exhibited substantial variation, which may be related to the degree of divergence between the genotypes, with RNS.09 and VNI.02 being the most divergent. In this context, genotypes with high genotypic divergence can be considered better options for parental selection in breeding programs. This is because genotypes that exhibit greater dissimilarity can produce offspring with increased genetic variability and a higher heterotic effect^79^, leading to a phenotypic response in productivity above the population average and, consequently, greater selective gains. A comprehensive understanding of the degree of divergence among accessions requires genotype analyses based on morpho-agronomic and molecular data^80^. This allows conclusions about the genetic divergence among the analyzed genotypes and supports decision-making regarding controlled crossing strategies, field organization, and conservation population management.

The Mantel test, performed between the distance matrices calculated from genotypic and molecular data, revealed an absence of correlation between them. A similar pattern was observed in the characterization of *E. oleracea* accessions based on morpho-agronomic and molecular traits^81^. According to the authors, the lack of correlation is due to the fact that morpho-agronomic traits are controlled by multiple genes and are influenced by environmental factors, unlike genetic markers, which are associated with and distributed throughout the genome. In the present study, data correction and the use of genetic values for diversity analysis may explain the observed differences, as the high number of genes controlling quantitative traits enhances the capture of diverse genetic effects, leading to results distinct from those obtained with a limited number of microsatellite markers.

The different classifications and lack of association between the distance matrices highlight the necessity of integrating morphometric and molecular data to enhance the discriminatory power of genotypes, thereby generating more accurate differentiation results. To achieve this, dissimilarity indices can be used in combination with genotype dissimilarity analyses^82^, as applied in the present study and illustrated in Fig. 6, with the aim of improving the efficiency of genotype characterization and differentiation^82^.

Regarding cluster analysis based on both data types, no grouping by geographic distribution was observed. Genotypes sampled from distant locations appeared within the same branches (Fig. 6). Marçal et al.^83^ reported similar results when evaluating the genetic diversity of *E. edulis* in forest fragments in Espírito Santo. According to the authors, the random distribution of origins within groups suggests the presence of genetic diversity among the accessions. The mixture observed in this study confirms the efficiency of the sampling strategy in capturing genetic diversity within and between municipalities, indicating the sampled matrices’ capacity to support the conservation of the species’ genetic resources in an *ex-situ* germplasm bank.

Although the groups were not defined by geographic proximity, the purple and orange clusters (Fig. 6B) were the largest and stood out due to their constituent genotypes. In the purple cluster, genotypes originated from different regions, whereas the orange cluster predominantly grouped genotypes from Venda Nova do Imigrante. This finding may serve as preliminary evidence of limited genetic variability in the forest fragments of this region, leading to the hypothesis of a high inbreeding coefficient or a founder effect resulting from a small sample size. The differentiation of this group from others underscores the distinctiveness of this locality, emphasizing the importance of sampling VNI genotypes to maximize genetic diversity in the breeding founders populations and germplasm collection.

The genetic and molecular diversity analyses revealed substantial divergence among genotypes from different collection sites, supporting the recommendation to prioritize sampling across multiple geographic regions. By combining genotypes from distinct genetic groups in germplasm collections, aligning their spatial arrangement in the field based on their genetic distance estimates, and placing genetically more distant genotypes in closer proximity, it is possible to enhance the potential for genetic recombination and broaden the genetic base of breeding populations. This approach not only fosters the development of improved genotypes but also contributes to the long-term conservation of genetic resources in *E. edulis*. The intrapopulational diversity of *E. edulis* has been documented in studies of natural populations, where it has been linked to factors such as reproductive system function, pollen and seed dispersal patterns, forest fragmentation and conservation status, and geographic origin^55^. Genetic diversity across different geographic regions implies varying adaptive capacities among genotypes, which is crucial for breeding programs^83^ and the conservation of *E. edulis*.

The results obtained in this study can serve as a foundation for breeding and conservation programs. By selecting more divergent genotypes for crossing, it is possible to maximize the genetic diversity of future progenies while minimizing inbreeding depression. Consequently, these breeding foundation population will play a crucial role in supporting *E. edulis* conservation, preservation and breeding programs by providing seeds with high genetic variability to aid in the enrichment of forest fragments. Moreover, they will serve as genetic reservoirs that can be leveraged for breeding programs. The results of this study provide valuable insights into genetic control and trait associations, while also offering relevant information on the diversity of the sampled population. These findings are crucial for supporting the structuring and optimization of breeding and conservation programs for the species.

## Conclusions

Emergence and morphometric traits of fruits and seeds exhibited high $H^2$ , whereas seedling traits had relatively smaller $H^2$ estimates. The significant positive $r_g$ between the morphometric characteristics of fruits and seeds and those related to growth indicates the potential for using these descriptors for early indirect selection to develop higher-quality seedlings. For seedling selection targeting quality (DQI), indirect selection can be primarily based on LA and DBS. Path analysis confirmed that LA and DSB exert the strongest direct effects on DQI, reinforcing their role as reliable early indicators of seedling quality. The detection of significant genetic variability for all traits, especially fruit and seed biometric characteristics, highlights the existence of broad genetic diversity among the *E. edulis* genotypes evaluated. Moreover, the low significance of genotype $\times$ time and genotype $\times$ environment interactions suggests phenotypic stability under the conditions tested, which is advantageous for selection.

The high magnitudes of the genetic diversity index estimates from the molecular markers may have been a direct response to the sampling method employed. This confirms the effectiveness of the sampling strategy used in this study in maximizing the capture of genetic variability for the establishment of a robust breeding founding population for genetic breeding programs and construction of germplasm in genetic conservation programs. Additionally, the high polymorphism, heterozygosity, and PIC values across loci reinforce the usefulness of these markers for characterizing genetic resources in *E. edulis*. The absence of correlation between genetic and molecular distance matrices (Mantel test) suggests that neutral markers alone may not fully capture functional genetic variation relevant to phenotypic performance, underscoring the importance of integrative approaches. The diversity analysis, using molecular and genotypic information, provided insights-via the dendrogram-on how to guide the establishment of breeding orchards of an *ex-situ* to maximize crosses between the most divergent genotypes. The grouping patterns identified offer a practical framework for crossing strategies aimed at maximizing heterosis and genetic gain.

## Supplementary Information

Supplementary Information.

## Data Availability

The phenotypic data generated and analyzed during the current study are available from the corresponding author upon reasonable request. The microsatellite marker datasets are publicly available at: https://github.com/GuilhermeBravim/-Divergence-Patterns-in-Euterpe-edulis-for-Breeding-and-Conservation-Applications.

## Declarations

### Permission to collect

The authors declare that all collections of *E. edulis* were conducted on private properties with prior authorization from the respective landowners. Furthermore, all sampling activities were formally authorized by the Brazilian Ministry of the Environment through the Chico Mendes Institute for Biodiversity Conservation (ICMBio), under the SISBIO system. It is important to emphasize that these activities were carried out exclusively for scientific purposes.
