# Supplementary material for: Uncovering morphometric, germination, and genetic divergence patterns in Euterpe edulis for breeding and conservation applications
Source: Sci Rep. 2025 Sep 26;15:33038. doi: 10.1038/s41598-025-02606-7 (PMC12474960; doi:10.1038/s41598-025-02606-7)
Supplement: Supplementary file 1 — Supplementary Information. [file 41598_2025_2606_MOESM1_ESM.pdf]

Uncovering Morphometric, Germination, and Genetic Divergence Patterns in *Euterpe edulis* for Breeding and Conservation Applications

GUILHERME BRAVIM CANAL[1], MARCELLO ZATTA PÉRES[2], FRANCINE ALVES NOGUEIRA DE ALMEIDA[1], MAURÍCIO DOS SANTOS ARAÚJO[3], JÉSSICA TETZNER DE OLIVEIRA[2], JÔNATAS GOMES SANTOS[1], GILZA BARCELOS DE SOUZA[1], MARCIA FLORES DA SILVA FERREIRA[1], RODRIGO SOBREIRA ALEXANDRE[2], AND ADÉSIO FERREIRA[1,*]

[1] *Department of Agronomy, Federal University of Espírito Santo, Alegre, Espírito Santo, Brazil*
[2] *Department of Forest and Wood Sciences, Federal University of Espírito Santo, Jerônimo Monteiro, Espírito Santo, Brazil*
[3] *Genetics Department, University of São Paulo, Genetic Diversity and Plant Breeding Laboratory, Piracicaba - SP, Brazil*
[*] *adesioferreira@gmail.com*

Euterpe edulis exhibits significant ecological, social, and economic potential, comparable to *E. oleracea*, for both the fresh fruit pulp market and industrial applications as a natural pigment. The commercial management of *E. edulis* fruits is economically promising; however, further studies are required to support improvement, conservation, and propagation programs. In this study, we aimed to assess the genetic control of key traits by estimating variance components, genetic parameters, and the associative relationships between fruit morphometric traits, seed characteristics, and seedling emergence using Pearson correlation and path analysis. Additionally, we quantified the genetic divergence among *E. edulis* accessions. Thirteen phenotypic traits and eight microsatellite markers were analyzed in 72 genotypes from different origins. High heritability values (ranging from 0.75 to 0.99) were observed for fruit, seed, and emergence traits, whereas morphophysiological growth traits exhibited lower heritability estimates. Genetic correlations of up to 0.88 were detected among fruit morphometric traits. Seedling basal diameter and leaf area had direct positive effects on the seedling quality index, while seedling height exerted a direct negative effect. Genetic divergence analysis demonstrated the efficiency of sampling genotypes from different origins to preserve the species' genetic variability. These findings provide insights to guide the establishment of germplasm collections in the field, maximizing the potential for genetic recombination among divergent genotypes and offering a solid foundation for future studies employing expanded molecular resources to explore trait architecture more deeply.

Table S1. Likelihood Ratio Test (LRT) with significance tested by the χ^2 test; Log-likelihood (LL), Akaike Information Criterion (AIC), and Bayesian Information Criterion (BIC) for each model for equatorial diameter fruit (EDF) (mm), weight of 25 fruits (WF) and seed (WS) (g), emergence speed index (ESI), mean emergence time (MET), percentage of emergence (E), diameter at stem height (DSB) (mm), seedling height (SH), leaf area (LA) (cm²), shoot dry mass (SDM) (g), total dry mass (TDM) (g), Dickson's quality index (DQI), and variable fluorescence/maximal fluorescence (FV/FM). Additionally, results are provided for comparison between the selected model and the model with the fixed effects of collection (β_c) and different measurement times (β_m; β_h).

Trait	Effects tested	LL	LRT	AIC	BIC	LL	AICc	BICc
EDF	ϕ	-497.65	–	997.30	1001.87	–	–	–
	σ^2_g	-42.799	909.70 ***	89.60	98.74	-40.77	87.54	101.25
	$\sigma^2_g + \sigma^2_{al}$	-42.799	-3.53×10^{-6} ns	91.60	105.31	–	–	–
	$\beta_c + \sigma^2_g$	–	–	–	–	-39.76	89.53	112.38
WF	ϕ	-785.40	–	1572.80	1576.45	–	–	–
	σ^2_g	-361.70	847.39 ***	727.41	734.72	-361.80	729.60	740.58
	$\sigma^2_g + \sigma^2_{al}$	-361.70	-2.00×10^{-4} ns	729.41	740.37	–	–	–
	$\beta_c + \sigma^2_g$	–	–	–	–	-359.29	728.58	746.87
WS	ϕ	-694.11	–	1390.22	1393.88	–	–	–
	σ^2_g	-313.15	761.91 ***	630.31	637.61	-314.27	638.54	656.86
	$\sigma^2_g + \sigma^2_{al}$	-313.15	-8.06×10^{-6} ns	632.31	643.27	–	–	–
	$\beta_c + \sigma^2_g$	–	–	–	–	-318.44	642.87	653.87
ESI	ϕ	397.90	–	-793.80	-790.21	–	–	–
	σ^2_g	516.42	237.05 ***	-1028.85	-1021.68	–	–	–
	$\sigma^2_g + \sigma^2_{al}$	519.03	5.21 *	-1032.06	-1021.31	523.18	-1038.37	-1024.02
	$\sigma^2_g + \sigma^2_{al} + \sigma^2_{gal}$	519.03	1.26×10^{-5} ns	-1030.06	-1015.73	–	–	–
	$\beta_c + \sigma^2_g + \sigma^2_{al}$	–	–	–	–	547.24	-1082.47	-1060.95
MTE	ϕ	-829.10	–	1660.20	1663.75	–	–	–
	σ^2_g	-742.14	173.91 ***	1488.29	1495.39	-742.64	1491.29	1501.95
	$\sigma^2_g + \sigma^2_{al}$	-741.28	1.72 ns	1488.57	1499.22	–	–	–
	$\beta_c + \sigma^2_g$	–	–	–	–	-715.84	1441.68	1459.44
E	ϕ	-1015.76	–	2033.52	2037.11	–	–	–
	σ^2_g	-890.66	250.19 ***	1785.34	1792.50	–	–	–
	$\sigma^2_g + \sigma^2_{al}$	-887.92	5.49 **	1781.85	1792.60	-889.08	1786.16	1800.51
	$\sigma^2_g + \sigma^2_{al} + \sigma^2_{gal}$	-887.92	1.05×10^{-6} ns	1783.85	1798.18	–	–	–
	$\beta_c + \sigma^2_g + \sigma^2_{al}$	–	–	–	–	-864.88	1750.44	1771.97
DSB	ϕ	-4474.29	–	8950.58	8956.98	–	–	–
	σ^2_g	-4282.59	383.41 ***	8569.18	8581.97	–	–	–
	$\sigma^2_g + \sigma^2_m$	-1320.33	5924.50 ***	2646.67	2665.86	–	–	–
	$\sigma^2_g + \sigma^2_m + \sigma^2_{gm}$	-821.64	997.38 ***	1651.29	1676.88	-821.16	1652.32	1684.32
	$\beta_m + \sigma^2_g + \sigma^2_{gm}$	–	–	–	–	-801.70	1621.41	1678.99
SH	ϕ	-10029.06	–	20060.12	20066.51	–	–	–
	σ^2_g	-9748.39	561.34 ***	19500.78	19513.56	–	–	–
	$\sigma^2_g + \sigma^2_m$	-7358.08	4780.60 ***	14722.15	14741.31	–	–	–
	$\sigma^2_g + \sigma^2_m + \sigma^2_{gm}$	-6998.84	718.48 ***	14005.67	14031.22	-6999.59	14009.18	14041.12
	$\beta_m + \sigma^2_g + \sigma^2_{gm}$	–	–	–	–	-6980.56	13979.11	14036.59
LA	ϕ	-7463.91	–	14929.83	14935.33	–	–	–

(Continued on next page)

(Continued from previous page)

Trait	Effects tested	LL	LRT	AIC	BIC	LL	AICc	BICc
	σ_g^2	-7383.56	160.71 ***	14771.12	14782.11	–	–	–
	$\sigma_g^2 + \sigma_m^2$	-6789.97	1187.20 ***	13585.94	13602.42	–	–	–
	$\sigma_g^2 + \sigma_m^2 + \sigma_{gm}^2$	-6676.45	227.04 ***	13360.89	13382.87	-6679.12	13368.24	13395.71
	$\beta_m + \sigma_g^2 + \sigma_{gm}^2$	–	–	–	–	-6667.84	13349.68	13388.15
SDM	ϕ	2228.71	–	-4455.43	-4449.66	–	–	–
	σ_g^2	2307.77	158.12 ***	-4611.54	-4600.01	–	–	–
	$\sigma_g^2 + \sigma_m^2$	2970.55	1325.60 ***	-5935.10	-5917.80	–	–	–
	$\sigma_g^2 + \sigma_m^2 + \sigma_{gm}^2$	3088.33	235.56 ***	-6168.66	-6145.59	3090.93	-6171.86	-6143.01
	$\beta_m + \sigma_g^2 + \sigma_{gm}^2$	–	–	–	–	3104.97	-6193.94	-6147.79
TDM	ϕ	-451.30	–	904.60	910.41	–	–	–
	σ_g^2	-367.87	166.86 ***	739.74	751.37	–	–	–
	$\sigma_g^2 + \sigma_m^2$	605.86	1947.50 ***	-1205.71	-1188.28	–	–	–
	$\sigma_g^2 + \sigma_m^2 + \sigma_{gm}^2$	736.72	261.73 ***	-1465.44	-1442.20	738.07	-1466.15	-1437.09
	$\beta_m + \sigma_g^2 + \sigma_{gm}^2$	–	–	–	–	753.18	-1490.37	-1443.87
DQI	ϕ	3535.29	–	-7068.58	-7062.89	–	–	–
	σ_g^2	3630.46	190.34 ***	-7256.92	-7245.54	–	–	–
	$\sigma_g^2 + \sigma_m^2$	4490.34	1719.80 ***	-8974.69	-8957.62	–	–	–
	$\sigma_g^2 + \sigma_m^2 + \sigma_{gm}^2$	4703.16	425.63 ***	-9398.31	-9375.56	4706.33	-9402.65	-9374.20
	$\beta_m + \sigma_g^2 + \sigma_{gm}^2$	–	–	–	–	4720.63	-9425.27	-9379.75
FV/FM	ϕ	981.04	–	-1960.08	-1955.91	–	–	–
	σ_g^2	999.71	37.34 ***	-1995.42	-1987.08	–	–	–
	$\sigma_g^2 + \sigma_h^2$	1040.31	81.20 ***	-2074.62	-2062.10	1043.86	-2079.72	-2063.03
	$\sigma_g^2 + \sigma_h^2 + \sigma_{gh}^2$	1040.31	–	-2072.62	-2055.93	–	–	–
	$\beta_h + \sigma_g^2$	–	–	–	–	1048.30	-2088.60	-2071.91

Legend: ϕ: Reduced model; σ_g^2: Genetic variance; σ_m^2: Random evaluations effect variance; σ_{al}^2: Random sampling elevation effect variance; σ_h^2: Hourly measurement effect variance; σ_{gal}^2: Random genotype × height interaction effect variance; σ_{gm}^2: Genotype × evaluation interaction effect variance; σ_{gh}^2: Genotype × hour interaction effect variance.

Table S2. Covariance structures used to model the random effects G and R for the traits equatorial diameter of the fruit (EDF) (mm), weight of 25 fruits (WF) and seed (WS) (g), emergence speed index (ESI), mean emergence time (MET), percentage of emergence (E), shoot dry mass (SDM) (g), total dry mass (TDM) (g), Dickson's quality index (DQI), leaf area (LA) (cm^2), diameter at stem height (DSB) (mm), seedling height (SH) (cm), and variable fluorescence/maximal fluorescence (FV/FM). The tested structures were compound symmetry (CS), heterogeneous compound symmetry (HCS), first-order autoregressive (AR), and diagonal (DIAG). Additionally, statistical information is provided, including log-likelihood (LL), Akaike information criterion (AIC), and Bayesian information criterion (BIC) for each model and each trait.

Trait	G	Acronym	R	Acronym	LL	AIC	BIC
ESI	$J\sigma_g^2 + \sigma_{gc}^2 I_c \otimes I_f$	CS	$\sigma_e^2 I_n$	IDV	535.43	-1064.87	-1054.14
	$J\sigma_g^2 + \sigma_{gc}^2 I_c \otimes I_f$	CS	$D \otimes I_n$	DIAG	544.54	-1079.09	-1061.21
MET	$J\sigma_g^2 + \sigma_{gc}^2 I_c \otimes I_f$	CS	$\sigma_e^2 I_n$	IDV	-713.77	1431.53	1438.61
	$J\sigma_g^2 + \sigma_{gc}^2 I_c \otimes I_f$	CS	$D \otimes I_n$	DIAG	-707.53	1423.06	1437.23
E	$J\sigma_g^2 + \sigma_{gc}^2 I_c \otimes I_f$	CS	$\sigma_e^2 I_n$	IDV	-893.611	1793.222	1803.995
	$J\sigma_g^2 + \sigma_{gc}^2 I_c \otimes I_f$	CS	$D \otimes I_n$	DIAG	-732.868	1475.735	1493.5
DSB	$J\sigma_g^2 + \sigma_{gm}^2 I_t \otimes I_i$	CS	$\sigma_e^2 I_n$	IDV	-816.199	1638.397	1657.59
	$\sqrt{D}[I_t + \rho(J - I_t)]\sqrt{D} \otimes I_i$	HCS	$\sigma_e^2 I_n$	IDV	-761.858	1539.716	1590.896
	$\sigma_g^2 I_t + \Sigma \otimes I_i$	AR	$\sigma_e^2 I_n$	IDV	-810.355	1628.709	1654.299
	$\sqrt{D}[I_t + \rho(J - I_t)]\sqrt{D} \otimes I_i$	HCS	$D \otimes I_n$	DIAG	-150.116	326.2325	409.4001
SH	$J\sigma_g^2 + \sigma_{gm}^2 I_t \otimes I_i$	CS	$\sigma_e^2 I_n$	IDV	-6987.22	13980.45	13999.6
	$\sqrt{D}[I_t + \rho(J - I_t)]\sqrt{D} \otimes I_i$	HCS	$\sigma_e^2 I_n$	IDV	-6962.51	13941.02	13992.1
	$\sigma_g^2 I_t + \Sigma \otimes I_i$	AR	$\sigma_e^2 I_n$	IDV	-6969.03	13946.05	13971.59
	$\sigma_g^2 I_t + \Sigma \otimes I_i$	AR	$D \otimes I_n$	DIAG	-6596.09	13210.19	13267.65
LA	$J\sigma_g^2 + \sigma_{gm}^2 I_t \otimes I_i$	CS	$\sigma_e^2 I_n$	IDV	-6664.18	13334.36	13350.84
	$\sqrt{D}[I_t + \rho(J - I_t)]\sqrt{D} \otimes I_i$	HCS	$\sigma_e^2 I_n$	IDV	-6606.43	13224.85	13257.81
SDM	$J\sigma_g^2 + \sigma_{gm}^2 I_t \otimes I_i$	CS	$\sigma_e^2 I_n$	IDV	3083.942	-6161.89	-6144.58
	$\sqrt{D}[I_t + \rho(J - I_t)]\sqrt{D} \otimes I_i$	HCS	$\sigma_e^2 I_n$	IDV	3151.944	-6289.89	-6249.52
TDM	$J\sigma_g^2 + \sigma_{gm}^2 I_t \otimes I_i$	CS	$\sigma_e^2 I_n$	IDV	737.3079	-1468.62	-1451.19
	$\sqrt{D}[I_t + \rho(J - I_t)]\sqrt{D} \otimes I_i$	HCS	$\sigma_e^2 I_n$	IDV	806.4922	-1598.98	-1558.32
DQI	$J\sigma_g^2 + \sigma_{gm}^2 I_t \otimes I_i$	CS	$\sigma_e^2 I_n$	IDV	4696.482	-9386.96	-9369.9
	$\sqrt{D}[I_t + \rho(J - I_t)]\sqrt{D} \otimes I_i$	HCS	$\sigma_e^2 I_n$	IDV	4775.992	-9537.99	-9498.17
FV/FM	$J\sigma_g^2 + \sigma_{gm}^2 I_t \otimes I_i$	CS	$\sigma_e^2 I_n$	IDV	1037.942	-2071.88	-2063.55
	$J\sigma_g^2 + \sigma_{gm}^2 I_t \otimes I_i$	CS	$D \otimes I_n$	DIAG	1039.226	-2072.45	-2059.94

Legend: σ_g^2: genetic variance; σ_{gc}^2: variance of the genotype × collection interaction; σ_{gm}^2: variance of the genotype × measurement interaction; σ_e^2: residual variance; I is an identity matrix with a dimension indicated by its subscript (c: number of collections; t: number of measurements; i: number of wild founders genotypes; n: number of observations); J is a matrix of 1's with dimension $c \times c$ (ESI, TEM, and E) or $t \times t$ (DSB, SH, LA, SDM, TDM, DQI, FV/FM); D is a diagonal matrix of dimension $t \times t$ with elements being σ_{gm}^2; Σ is an autocorrelation matrix of dimension $t \times t$; ρ is the correlation coefficient; $\otimes$ is the Kronecker product.

Table S3. Variance components for the traits equatorial diameter of fruit (EDF) (mm), weight of 25 fruits (WF), and seed weight (WS) (g) in *Euterpe edulis*.

Variance Component	EDF (mm)	WF (g)	WS (g)
σ_g^2	1.21 ± 0.21	87.11 ± 14.66	50.66 ± 8.54
σ_e^2	0.28 ± 0.02	1.08 ± 0.10	0.86 ± 0.08

σ_g^2: genetic variance; σ_e^2: residual variance.

Table S4. Variance components for the traits emergence speed index (ESI), mean emergence time (MET), and percentage of emergence (E) in *Euterpe edulis*.

Variance Component	ESI	MET	E
σ_{al}^2	0.003 ± 0.002	–	6.39 ± 13.66
σ_g^2	0.004 ± 0.002	65.29 ± 14.67	58.66 ± 19.51
σ_{ec1}^2	0.004 ± 0.001	60.32 ± 13.06	60.34 ± 13.07
σ_{ec2}^2	0.005 ± 0.001	44.73 ± 6.33	44.09 ± 6.33
σ_{ec3}^2	0.002 ± 0.000	103.77 ± 20.86	104.1 ± 20.94

σ_{al}^2: variance of the random sampling elevation effect; σ_g^2: genetic variance; σ_{ec1}^2: residual variance for collection 1; σ_{ec2}^2: residual variance for collection 2; σ_{ec3}^2: residual variance for collection 3.

Table S5. Variance components for the variable fluorescence/maximal fluorescence (FV/FM) in *Euterpe edulis*.

Variance Component	FV/FM
σ_g^2	0.001 ± 0.0003
σ_{eh1}^2	0.004 ± 0.0004
σ_{eh2}^2	0.004 ± 0.0004

σ_g^2: genetic variance; σ_{eh1}^2: residual variance component for the first measurement time; σ_{eh2}^2: residual variance component for the second measurement time.

Table S6. Variance components for the traits diameter at stem height (DSB) (mm), seedling height (SH), leaf area (LA) (cm²), shoot dry mass (SDM) (g), total dry mass (TDM) (g), and Dickson's quality index (DQI) in *Euterpe edulis*.

Variance Component	DSB (mm)	SH	LA (cm²)	SDM (g)	TDM (g)	DQI
σ^2_g	0.12 ± 0.03	2.63 ± 1.33	104.83 ± 24.16	0.003 ± 0.001	0.02 ± 0.01	$0.001 \pm 1.71 \times 10^{-4}$
σ^2_{gm}	–	5.13 ± 1.21	–	–	–	–
σ^2_{g0}	0.02 ± 0.01	–	–	–	–	–
σ^2_{g50}	0.1 ± 0.03	–	–	0.001 ± 0.001	0.01 ± 0.004	$0 \pm 1.04 \times 10^{-4}$
σ^2_{g100}	0.1 ± 0.03	–	34 ± 16.49	0 ± 0	$4.56 \times 10^{-8} \pm 0$	$0 \pm 1.11 \times 10^{-4}$
σ^2_{g150}	0.63 ± 0.13	–	72.11 ± 22.54	0.004 ± 0.001	0.04 ± 0.01	$0.002 \pm 3.99 \times 10^{-4}$
σ^2_{g200}	0.56 ± 0.13	–	79.74 ± 28.42	0.01 ± 0.003	0.09 ± 0.02	$0.003 \pm 7.51 \times 10^{-4}$
σ^2_{g262}	0.8 ± 0.19	–	693.25 ± 166.17	0.024 ± 0.006	0.18 ± 0.05	$0.008 \pm 1.80 \times 10^{-4}$
σ^2_{e0}	0.18 ± 0.01	4.71 ± 0.15	–	–	–	–
σ^2_{e50}	0.3 ± 0.02	4.4 ± 0.28	–	0.008 ± 0	0.02 ± 0.002	$0.001 \pm 5.25 \times 10^{-4}$
σ^2_{e100}	0.31 ± 0.02	3.9 ± 0.27	105.87 ± 7.72	0.006 ± 0	0.04 ± 0.003	$0.001 \pm 6.15 \times 10^{-4}$
σ^2_{e150}	0.48 ± 0.03	6.99 ± 0.48	147.41 ± 10.45	0.014 ± 0.001	0.08 ± 0.01	$0.002 \pm 1.36 \times 10^{-4}$
σ^2_{e200}	0.91 ± 0.06	14.3 ± 1	349.14 ± 24.82	0.022 ± 0.002	0.14 ± 0.01	$0.004 \pm 2.96 \times 10^{-4}$
σ^2_{e262}	1.43 ± 0.1	21.61 ± 1.51	1326.7 ± 94.93	0.066 ± 0.005	0.59 ± 0.04	$0.013 \pm 9.27 \times 10^{-4}$

σ^2_g: genetic variance; σ^2_{gm}: variance of genotype × measurement interaction; σ^2_{g0}: genetic variance at the first measurement (day 0); σ^2_{g50}: genetic variance at the second measurement (day 50); σ^2_{g100}: genetic variance at the third measurement (day 100); σ^2_{g150}: genetic variance at the fourth measurement (day 150); σ^2_{g200}: genetic variance at the fifth measurement (day 200); σ^2_{g262}: genetic variance at the sixth measurement (day 262); σ^2_{e0}: residual variance at the first measurement (day 0); σ^2_{e50}: residual variance at the second measurement (day 50); σ^2_{e100}: residual variance at the third measurement (day 100); σ^2_{e150}: residual variance at the fourth measurement (day 150); σ^2_{e200}: residual variance at the fifth measurement (day 200); σ^2_{e262}: residual variance at the sixth measurement (day 262).
